# Oncofetal Chondroitin Sulfate Is a Highly Expressed Therapeutic Target in Non-Small Cell Lung Cancer

**DOI:** 10.3390/cancers13174489

**Published:** 2021-09-06

**Authors:** Htoo Zarni Oo, Zoltan Lohinai, Nastaran Khazamipour, Joey Lo, Gunjan Kumar, Jessica Pihl, Hans Adomat, Noushin Nabavi, Hakhamanesh Behmanesh, Beibei Zhai, Robert Dagil, Swati Choudhary, Tobias Gustavsson, Thomas M. Clausen, Jeffrey D. Esko, Jeffrey W. Allen, Michael A. Thompson, Nhan L. Tran, Judit Moldvay, Balazs Dome, Ali Salanti, Nader Al-Nakouzi, Glen J. Weiss, Mads Daugaard

**Affiliations:** 1 Department of Urologic Sciences, University of British Columbia, Vancouver, BC V6H 3Z6, Canada; hoo@prostatecentre.com (H.Z.O.); nkhazamipour@prostatecentre.com (N.K.); jlo@prostatecentre.com (J.L.); gkumar@prostatecentre.com (G.K.); hans.adomat@vch.ca (H.A.); nnabavi@prostatecentre.com (N.N.); hakhab@hotmail.com (H.B.); bzhai@prostatecentre.com (B.Z.); nalnakouzi@prostatecentre.com (N.A.-N.); 2 Vancouver Prostate Centre, Vancouver Coastal Health Research Institute, Vancouver, BC V6H 3Z6, Canada; 3 Department of Tumor Biology, National Koranyi Institute of Pulmonology, 1122 Budapest, Hungary; zoltan.lohinai@koranyi.hu (Z.L.); moldvay@koranyi.hu (J.M.); balazs.dome@meduniwien.ac.at (B.D.); 4 Department of Cellular and Molecular Medicine, University of California, La Jolla, San Diego, CA 92093, USA; jessica.pihl@sund.ku.dk (J.P.); tmandelclausen@health.ucsd.edu (T.M.C.); jesko@health.ucsd.edu (J.D.E.); 5 Department for Immunology and Microbiology, Faculty of Health and Medical Sciences, University of Copenhagen, 2200 Copenhagen, Denmark; robert@sund.ku.dk (R.D.); swati@sund.ku.dk (S.C.); tobias@sund.ku.dk (T.G.); salanti@sund.ku.dk (A.S.); 6 Kootenai Health, Post Falls, ID 83854, USA; jallen@kh.org; 7 Aurora Cancer Care, Advocate Aurora Health, Milwaukee, WI 53215, USA; Michael.Thompson2@aah.org; 8 Department of Cancer Biology, Mayo Clinic, Scottsdale, AZ 85259, USA; Tran.Nhan@mayo.edu; 9 MTA-SE NAP, Brain Metastasis Research Group, Department of Pathology, Hungarian Academy of Sciences, 1085 Budapest, Hungary; 10 Department of Thoracic Surgery, National Institute of Oncology, Semmelweis University, 1122 Budapest, Hungary; 11 Department of Thoracic Surgery, Medical University of Vienna, 1090 Vienna, Austria; 12 MiRanostics Consulting, Oro Valley, AZ 85755, USA; gweiss@intgen.org

**Keywords:** NSCLC, chondroitin sulfate, glycosaminoglycan, VDC-MMAE, drug conjugate

## Abstract

**Simple Summary:**

While molecular targeted therapy has marginally improved outcomes of patients with non-small cell lung cancer (NSCLC), NSCLC remains a significant health care challenge with a poor prognosis. We hypothesized that a broad-spectrum oncofetal chondroitin sulfate (CS) glycosaminoglycan modification, redundantly expressed on cell surface proteoglycans and previously described in other solid tumor indications, could offer a prognostic and therapeutic handle on NSCLC. Here, we report that elevated oncofetal CS expression predicts poor disease-free and overall survival in four independent patient cohorts of early-stage NSCLC (*n* = 493), independent of KRAS and EGFR mutations. Additionally, we show that a novel preclinical oncofetal CS-targeting drug conjugate effectively eliminates NSCLC cells in vitro and inhibits KRAS-mutated NSCLC xenograft tumor growth in vivo. These results provide clinical and preclinical proof that oncofetal CS is an actionable prognosticator and therapeutic receptor in wild-type and KRAS-mutated NSCLC.

**Abstract:**

Broad-spectrum therapeutics in non-small cell lung cancer (NSCLC) are in demand. Most human solid tumors express proteoglycans modified with distinct oncofetal chondroitin sulfate (CS) chains that can be detected and targeted with recombinant VAR2CSA (rVAR2) proteins and rVAR2-derived therapeutics. Here, we investigated expression and targetability of oncofetal CS expression in human NSCLC. High oncofetal CS expression is associated with shorter disease-free survival and poor overall survival of clinically annotated stage I and II NSCLC patients (*n* = 493). Oncofetal CS qualifies as an independent prognosticator of NSCLC in males and smokers, and high oncofetal CS levels are more prevalent in EGFR/KRAS wild-type cases, as compared to mutation cases. NSCLC cell lines express oncofetal CS-modified proteoglycans that can be specifically detected and targeted by rVAR2 proteins in a CSA-dependent manner. Importantly, a novel VAR2-drug conjugate (VDC-MMAE) efficiently eliminates NSCLC cells in vitro and in vivo. In summary, oncofetal CS is a prognostic biomarker and an actionable glycosaminoglycan target in NSCLC.

## 1. Introduction

Lung cancer is a fast-progressing tumor with low prevalence of oncogene-addicted subsets, and with each subset requiring unique therapies. Recent therapeutic improvements in advanced non-small cell lung cancer (NSCLC) include, but are not limited to, targeted therapies and checkpoint immunotherapy that have changed clinical practice. However, the 5-year survival of NSCLC is still ~16% and there is an unmet need for highly effective treatments to better outcomes for this cancer type [1]. In advanced NSCLC, the current standards of care are chemotherapy, immunotherapy, and targeted therapy in specific oncogene-addicted tumors. Available targeted therapies are mostly directed towards oncogene aberrations: EGFR, ALK, ROS1, BRAF, RET, MET [2], NTRK [3], and KRAS [4]. Targeted inhibitors have achieved good anti-tumor effects in patients with target-specific genomic alterations.

Currently, several antibody-drug conjugates (ADCs) are under clinical investigation with promising activity in advanced cancers [5]. Approved ADCs include trastuzumab emtansine that is a conjugation between a cytotoxic payload and an anti-HER2 antibody, targeting HER2 overexpressing cells. Other ‘smart’ medications that direct cytotoxic therapy to cancer cells by using a known biomarker include deruxtecan, trop-2 ADC in triple negative breast cancer [6], and nectin-4 ADC in urothelial cancer [7]. Surgery with or without adjuvant chemotherapy is regarded as the standard-of-care treatment for early-stage NSCLC, although only 25–30% of tumors are suitable for potentially curative resection. Additional treatment options for NSCLC are urgently needed to improve patient outcomes and quality of life. Although mutation-specific therapeutic targets offer the exciting prospect of efficacious therapies, broad-spectrum novel approaches are in demand.

Tumor cell surface glycosylation acts as key regulatory mechanisms controlling several pathophysiological processes, such as cell signaling, cell dissociation, cell-matrix interaction, angiogenesis, immune modulation, invasion, and metastasis [8]. Changes in glycosylation patterns are associated with carcinogenesis [9,10]. Specifically, chondroitin sulfate (CS) alterations are found in most solid tumors [11]. Modification of proteins with CS form biologically active molecules called chondroitin sulfate proteoglycans (CSPG) [12].

CS and CSPGs play crucial roles in malaria pathogenesis [13], and syndecan-1 (SDC1) is the major CSPG involved in pregnancy-associated *Plasmodium falciparum* malaria [14]. The parasitized erythrocytes can sequester in the placenta via specific adhesion to a CS signature expressed exclusively in the placental syncytium and intervillous space, mediated by the malaria-encoded protein VAR2CSA [15]. Many solid tumors express placental-type CS as a secondary oncofetal CS modification on proteoglycans, and the recombinant VAR2CSA malaria protein (rVAR2) can, therefore, also bind to malignant cells [16,17]. Moreover, oncofetal CS is important for cancer cell migration and can regulate metastasis via integrin signaling pathways [18]. Previous studies have shown that oncofetal CS is highly expressed in solid tumors such as melanoma and muscle-invasive bladder cancer, and is associated with advanced tumor stage and poor patient outcome [16,19]. However, its expression in lung cancer, especially in early-stage tumorigenesis, is poorly understood. This study aimed to analyze the expression of oncofetal CS in NSCLC and determine the susceptibility of NSCLC cells to oncofetal CS-targeting therapeutics.

## 2. Material and Methods

### 2.1. Lung Cancer Cohorts

In this retrospective study, surgically resected and histologically confirmed NSCLC tumor tissues were obtained, with the approval of the local institutional review board, from patients diagnosed with NSCLC with early-stage disease: 318 cases of stage I and 187 cases of stage II (according to the contemporary guidelines and AJCC 7th edition) [20]. No data were available for 34 cases. Patients were diagnosed between the years 2001 and 2013 and received follow-up care according to the contemporary NCCN guidelines at Scottsdale Healthcare, Scottsdale, AZ (SHC); ProHealth Care, Waukesha, WI (PHC); National Koranyi Institute of Pulmonology, Budapest; or University of Tennessee, Knoxville, TN (UTenn). SHC and UTenn cohort data without detailed prognostic or OS data were published before [16,21]. Clinicopathological data included gender, age, stage, molecular characteristics (EGFR and KRAS where available), smoking, disease-fee survival (DFS), and overall survival (OS). We assessed the pathological tumor (pT) and lymph node (LN) status according to the Union for International Cancer Control (7th edition), and recorded age at the time of diagnosis. Patients who underwent lung resection surgery including lobectomy or pulmonectomy were included. OS was estimated from the time of diagnosis, until death, or last available follow-up. The study and all treatments were conducted based on the institutional guidelines.

### 2.2. Tissue Micro Arrays (TMA)

NSCLC resected tumors were formalin fixed and paraffin embedded (FFPE) after surgery. TMA construction from FFPE blocks was performed. Briefly, from each tissue block, 4-micron sections were cut using a microtome and placed on slides. Slides were stained for H&E. Board-certified pathologists marked the tumor area on H&E slides. Two 1-mm punches of tissue were taken from each primary tumor tissue block for TMA construction. TMAs from four separate patient cohorts, with two cores from each patient, representing a total of 539 clinically annotated stage I–II NSCLC cases were examined. The TMAs were built with Veridiam Semi-Automated Tissue Arrayer (VTA-100, Veridiam, San Diego, CA, USA).

### 2.3. Cell Lines

HCC827, H358, H1975, H2030, H460, H520, A427, H1993, H2073, H1792, H2122, H23, H1703, and A549 NSCLC cell lines (ATCC) were grown in appropriate cell culture medium.

### 2.4. Protein Constructs

All recombinant rVAR2 proteins were expressed in *E. coli* Shuffle cells (NEB) using a pET28 vector. The constructs of rVAR2 (ID1-ID2a) contained either a SpyTag [22,23] or an albumin-binding domain (ABD) [24] for extension of plasma half-life fused in the N-terminal, and in the C-terminal a V5-tag and 6×His-tag were added for detection and affinity purification. The control proteins, DBL4 and DBL5, both contained a V5-tag and His-tag in the C-terminal similar to rVAR2 and for DBL5 an ABD was fused in the N-terminal.

Expression and purification of the rVAR2 protein were performed, as described in a previous publication [16]. Briefly, the *E. coli* pellet harboring the recombinant protein was resuspended in lysis buffer with benzonase and homogenized. After centrifugation and sterile-filtration, the supernatant was loaded onto a HisTrap HP (Cytiva, Uppsala, Sweden) column and eluted using imidazole. The protein was further purified by HiTrap SP HP (Cytiva, Uppsala, Sweden), before elution an endotoxin reduction wash step was included where 0.1% Triton X114 in binding buffer was passed over the column. After removing the wash buffer, protein was eluted with a linear gradient from 0 to 1 M NaCl in 25 mM phosphate buffer pH 7.2. The eluted rVAR2 protein was pooled and analyzed by SDS-PAGE, aliquoted, flash-frozen, and stored at −80 °C until use.

### 2.5. Immunohistochemistry (IHC)

Freshly cut TMA sections were analyzed for oncofetal CS expression and IHC was performed using the Ventana Discovery platform, as described previously [16]. Interpretation of all immunostainings was blinded from clinicopathological parameters and patient outcome. The intensity and percent positivity were examined for both pericellular and extracellular immunostaining. While an overall score was calculated for each tissue core, an average score of 100 to 300 was considered as oncofetal CS high. The data were analyzed for correlation between low and high oncofetal CS expression of both tumor and stroma cells, with respect to EGFR and KRAS mutations, as well as clinical characteristics including DFS and OS.

### 2.6. Flow Cytometry

Cells were grown to 70–80% confluency in appropriate growth media and harvested in an EDTA detachment solution (Cellstripper^®^, Mediatech, Inc., Manassas, VA, USA). Cells were incubated with rVAR2-V5-tagged protein (200 nM–25 nM) in PBS containing 2% FBS for 30 min at 4 °C and binding was analyzed in a FACSCalibur (BD FACSCanto II, Becton, Dickinson and Company, Franklin Lakes, NJ, USA) after secondary incubation with anti-V5-FITC antibody for 30 min at 4 °C (1:500, Invitrogen, Carlsbad, CA, USA; Cat#R963-25). For inhibition studies, the protein was co-incubated with 400 μg/mL CSA (Sigma, Saint Louis, MO, USA; Cat#27042). A recombinant V5-tagged non-CS binding domain of the VAR2CSA protein (DBL4) was used as a control, and results were analyzed using FlowJo software (version 10.1r7, Ashland, OR, USA). Mean fluorescence intensity (MFI) was plotted as a percentage of the highest MFI signal within the same cell line. Background was calculated from cells incubated with secondary anti-V5-FITC only and the signal was subtracted from each MFI.

### 2.7. Preparation of Drug Conjugate VDC-MMAE and Control-MMAE

We chemically conjugated a potent anti-mitotic agent, monomethyl auristatin E (MMAE), derived from peptides occurring in marine shell-less mollusk *Dolabella auricularia* to rVAR2 via a maleimide-activated cathepsin B valine-citrulline cleavable linker, utilizing free cysteines in the recombinant protein. Briefly, the rVAR2-drug conjugate (VDC) was obtained by mixing rVAR2 (ABD-ID1-ID2a) or control protein (ABD-DBL5) and the drug linker in a 1:2.8 molar excess and incubating for 60 min at room temperature. To concentrate and remove unreacted drug linker, the protein was then loaded on a HiTrap SP HP column equilibrated with binding buffer (25 mM sodium phosphate, pH 7.2) and eluted with 1 M NaCl in 25 mM sodium phosphate, pH 7.2. The eluted material was buffer exchanged into 20 mM Histidine, 200 mM NaCl, pH 6.0, flash-frozen, and stored at −80 °C prior to use. Purity and Drug-VAR2 Ratio (DVR) was determined by SDS-PAGE, HPLC-SEC, and UV-vis absorbance measurement. The rVAR2-MMAE drug conjugate (VDC-MMAE) had a DVR of 1.8, whereas the DBL5-MMAE (Control-MMAE) had a DVR of 2.3.

### 2.8. In Vitro Cytotoxicity Assay of VDC-MMAE of Human Lung Cancer Cell Lines

Cells were removed from their culture vessel using Corning™ CellStripper Dissociation Reagent (Mediatech, Inc., Manassas, VA, USA; #MT25056CI). Detached cells were diluted in respective growth medium (Thermo Fisher Scientific, Waltham, MA, USA; #11095-080) + 10% Fetal bovine serum (Corning; #35-015-CV) to 25,000 cells/mL such that 100 µL/well would dispense 2500 cells/well. Cells were seeded into black-walled, clear, flat-bottomed, 96-well plates (Corning™ Costar™, Fisher Scientific, Waltham, MA, USA; #3595). Cells were incubated for one night at 37 °C in a 5% CO_2_ atmosphere to allow the cells to attach to the microtiter plate surface. VDC-MMAE was diluted directly in the appropriate cell growth medium and then titrated 1:2, over nine steps starting at 300 nM. A control with no VDC-MMAE present (growth medium alone) was included on each microtiter plate in triplicates. Then, 400 μg/mL CSA was used as a specificity control as well as a toxicity rescue assay in three of the 14 cell lines tested. Then, 50 µL/well of the prepared VDC-MMAE titrations was added in triplicate to each cell line assayed. The cells and titrations were incubated at 37 °C with 5% CO_2_ for 48 hours. After the incubation, cell viability was measured by using a crystal violet proliferation assay. The collected relative light absorbance [LA] was converted to % cytotoxicity using the absorbance values measured from the growth medium alone control as follows:

% Cytotoxicity = 1 − [Well LA/average medium alone control LA](1)

Data (% Cytotoxicity vs. Concentration of VDC (log_10_[nM]) were plotted and were analyzed by non-linear regression methods using GraphPad Prism software version 8 (GraphPad Software, San Diego, CA, USA) to obtain IC50 estimates.

### 2.9. The rVAR2 Pulldown and Mass Spectrometry

Cells were homogenized and spun down, and membrane proteins were extracted by incubating the membrane pellet in EBC lysis buffer (20 mM NaCl, 50 mM Tris-HCl, 2.5 mM MgCl_2_, 1 mM EDTA) supplemented with a protease inhibitor cocktail (Roche Diagnostics GmbH, Mannheim, Germany). CS proteoglycan enrichment was done using an rVAR2 affinity chromatography workflow, as previously described [25]. Briefly, Spy-tagged rVAR2 protein was loaded onto a SpyCatcher modified HiTrap Sepharose column (GE Healthcare, Chicago, IL, USA), which allowed immobilization of rVAR2 through isopeptide formation between the tag-catcher system. The extracted samples were then loaded onto the column and washed extensively (0.25 M NaCl), and bound proteins were eluted using high-salt buffer (1.6 M NaCl). The samples were desalted using PD10 columns (GE Healthcare, Sigma) and reduced with DTT (5 mM), alkylated with iodoacetamide (15 mM), and digested with trypsin 1:40 *w*/*w* (Promega, Madison, WI, USA) overnight. Finally, detergent was removed from the digested samples by Pierce detergent removal spin columns (Thermo Scientific, Waltham, MA, USA) and the peptides were desalted using C18 spin columns (Thermo Scientific). Samples were dried and stored at −20 °C until LC-MS/MS analysis.

Analysis of peptides was carried out on an Orbitrap Fusion Lumos MS platform (Thermo Scientific). Samples were introduced using an Easy-nLC 1200 system with an ES801, 50 µm ID × 15 cm column (Thermo Scientific). Prior to each sample injection, the analytical column was equilibrated at 500 bar, for a total volume of 10 μL. After injection, sample loading was carried out, for a total volume of 6 μL at 500 bar. The injection volumes for all samples were 2 µL, and were run using a gradient of mobile phase A (water and 0.1% formic acid) and B (80% acetonitrile with 0.1% formic acid) at 0.3 µL/min, 2–28% B from 2–92 min followed by 28–40% B, 92–102 min; 40–95% B, 102–108 min; 95%, 108–120 min; 50 °C.

Data acquisition (control software version 3.1.2412.17, Thermo Scientific, Waltham, MA) was carried out using a data-dependent method with MS2 in the Orbitrap. The Lumos was operated with a positive ion spray voltage of 2000 and a transfer tube temperature of 325 °C. The default charge state was set as 2. Survey scans (MS1) were acquired in the Orbitrap at a resolution of 60 K, across a mass range of 375–1500 *m*/*z*, with RF lens setting of 30, an AGC target of 4 × 10^5^, and max injection time of 50 ms in profile mode. For MS2 scans, an intensity threshold of 5 × 10^4^, charge state filtering of 2–5, and dynamic exclusion for 15 seconds with 10 ppm tolerances were used with a 1.2-*m*/*z* window prior to HCD fragmentation of 33%. MS2 data acquisition, carried out in the Orbitrap, used a 15-K resolution, a fixed first mass of 110 *m*/*z*, an AGC target of 5 × 10^4^, and a max injection time of 100 ms in centroid mode with parallelizable time turned off.

### 2.10. Mass Spectrometry Data Analysis

All data files were processed with Protein Discoverer 2.2.0.388 (Thermo Scientific, Waltham, MA, USA). Spectrum files were recalibrated and features extracted with Minora. Searches were carried out with Sequest HT with SwissProt TaxID = 9606 (v2017-10-25, Swiss Institute of Bioinformatics, Lausanne, Switzerland) with precursor mass tolerance 10 ppm and fragment mass tolerance 0.01 Da with C carbamidomethyl as permitted and fixed, and M, P oxidation, as permitted dynamic peptide modifications and acetyl N-terminal protein modification. Decoy database strict and relaxed FDR targets were 0.01 and 0.05 based on q value. Precursor quantification was intensity based with unique and razor peptides used, normalizing on total peptide amount with scaling on all average, and protein lists exported to Excel for any further analysis. The CSPG hits were verified against control pulldown samples from cell media.

### 2.11. In Vivo Study

All animal work was approved by the animal ethics application (A18-0278) and biosafety application (B19-0104) Animal Care Committee of the University of British Columbia. Initially, 7-week-old female nude mice (ENVIGO, Indianapolis, IN, USA; Stock No. 069) were anesthetized with 3% isoflurane. Then, 100 μL of a cell suspension in PBS:Matrigel (Corning, Ref. No. 354234) in 1:1 ratio, containing 1.0 × 10^6^ A549 cells, were injected into a total of 23 mice using a 27-G needle by subcutaneous injection. Starting on day 2, tumor volume was measured by caliper three times a week. Mice with growing tumor size between 120 mm^3^ to 150 mm^3^ were assigned to the following treatment groups: Vehicle (His-buffer; 20 mM His, 200 mM NaCl, pH 6.0) (*n* = 9), VDC-MMAE (*n* = 7) at 15 mg/kg, and Control-MMAE (*n* = 7) at 8.9 mg/kg, using sequential randomization. The VDC-MMAE dose was selected based on a previous dose-escalation study in healthy female CD-1 mice, which was well tolerated by the animals, without signs of morbidity or physical distress [16]. Treatments were administered twice a week for two weeks by intravenous injection in the tail vein. Survival was determined by reaching either experimental (tumor volume between 800–1000 mm^3^) or humane endpoints (such as weight loss >15%, tumor with a visible ulceration, which did not heal with metacam and polysporin treatments, irregular/labored respirations, ulcerated skin >1 cm patch, no response when stimulated, immobile, constantly shaking, vocalizations, and severe self-mutilation/trauma). The difference in tumor sizes between the two groups were analyzed using Student’s *t*-test.

### 2.12. Statistical Analyses

Statistical analyses were conducted using the PASW Statistics 22.0 package (SPSS Inc., Chicago, IL, USA). OS was calculated from the date of surgery to the date of death. Patients still alive were censored at the date of last follow-up. DFS was calculated from surgery until disease recurrence. To identify independent prognostic factors, Cox proportional hazards models were applied.

We used average *H*-score from TMA core A and B values, including both stroma and tumor compartments in the statistical analyses. Statistical analyses were performed only in patients with available clinicopathological data. Next, patients were divided into “high” (≥100) and “low” (<100) groups, based on the oncofetal CS expression averaged of both stroma and tumor tissue. According to low vs high oncofetal CS expression scores, clinicopathological characteristics of patients were analyzed by the Chi-square tests.

Kaplan–Meier curves and two-sided log-rank tests were used for univariate survival analysis. Two-sided *p*-values less than 0.05 were considered statistically significant. All variables with *p*-values less than 0.05 were included in the multivariate analysis. The Cox proportional hazards model was used for univariate and multivariate survival analyses to calculate the hazard ratios (HR) and corresponding 95% confidence intervals (CI). For multivariate survival analyses, the Cox regression model was adjusted for significant variables in the univariate analysis. Metric data are shown as median or mean and corresponding range or, in the case of OS, as median and corresponding 95% CI. We included gender, age, oncofetal CS expression, histology, and tumor stage in the Cox model.

## 3. Results

### 3.1. Baseline Clinical Characteristics and Patient Outcomes

We identified a total of 539 patients diagnosed with NSCLC who underwent surgical lung resection. The mean age at diagnosis was 65.6 ± 9.8 years. The median overall survival (OS) of the cohort was 66.7 ± 4.8 years. The clinicopathological characteristics of surgically resected NSCLC patients are outlined according to available low vs. high oncofetal CS expression in both tumor and stroma as average *H*-score in Table 1. Figure S1A represents the study design flow chart, including case numbers for this retrospective analysis with inclusion and exclusion criteria.

### 3.2. Oncofetal CS Expression Is an Independent Prognostic Classifier in Early-Stage NSCLC

**Figure 1:**
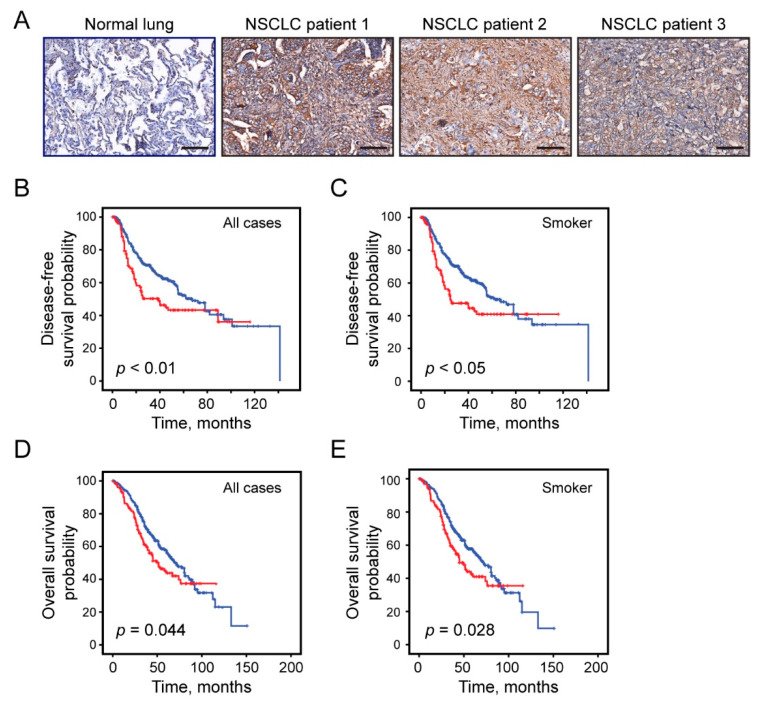
Oncofetal CS expression in NSCLC and survival analyses: (**A**) representative images of oncofetal CS expression in normal human lung and NSCLC tissue samples. The scale bar represents 100 μm. (**B**) Survival estimates of disease-free survival (DFS) in all cases and (**C**) smokers. (**D**) Estimate of overall survival (OS) in all early-stage NSCLC patients and (**E**) smokers. Red color bar: high oncofetal CS expression; blue: low expression. Patients were divided into “high” (≥100) and “low” (<100) groups based on the expression of oncofetal CS average stroma and tumor *H*-score.

We examined four cohorts of early-stage NSCLC samples as well as normal lung tissues for basal oncofetal CS expression using rVAR2 IHC. Oncofetal CS expression was observed in lung cancer tissue but was minimal or absent in normal lung (Figure 1A). There were 351 patients (71%) with low and 142 patients (29%) with high oncofetal CS expression. High oncofetal CS levels were associated with shorter DFS in all cases (39 vs. 67 months, *p* < 0.01) (Figure 1B) and smokers (*p* < 0.05) (Figure 1C). A similar prognosis stratification was also seen in OS for all cases (51 vs. 69 months, *p* = 0.044) (Figure 1D) and smokers (*p* = 0.028) (Figure 1E). Moreover, high oncofetal CS expression was associated with shorter DFS (*p* = 0.009) and OS (*p* < 0.01) in male patients (Figure S1B,C). There was no significant DFS or OS difference based on oncofetal CS expression when stratifying by EGFR or KRAS mutation status as well as female and non-smokers. Prevalence of high oncofetal CS level was highest in EGFR wild-type/KRAS wild-type cases followed by in EGFR wild-type/KRAS mutation patient samples (Table S1). High oncofetal CS expression was seen more frequently in squamous cell carcinoma than adenocarcinoma (46% vs. 19%, Table 1) and associated with poor DFS in squamous cell carcinoma (*p* = 0.012) (Figure S1D). In univariate survival analyses, we observed significant shorter median OS/DFS in male as well as in stage I and II groups with high oncofetal CS expression (Table 2). In multivariate survival analyses, when Cox regression model was adjusted for oncofetal CS, histology (adenocarcinoma vs. squamous cell carcinoma), and stage (I vs. II), high oncofetal CS expression was significantly associated with shorter DFS (HR, 1.76; 95% CI, 1.32–2.48; *p* < 0.01) (Table 3). Taken together, high oncofetal CS expression is present in around 30% of early-stage NSCLC patients and is an independent prognostic factor for poor DFS in NSCLC patients.

### 3.3. NSCLC Cell Lines Are Sensitive to VDC-MMAE

**Figure 2:**
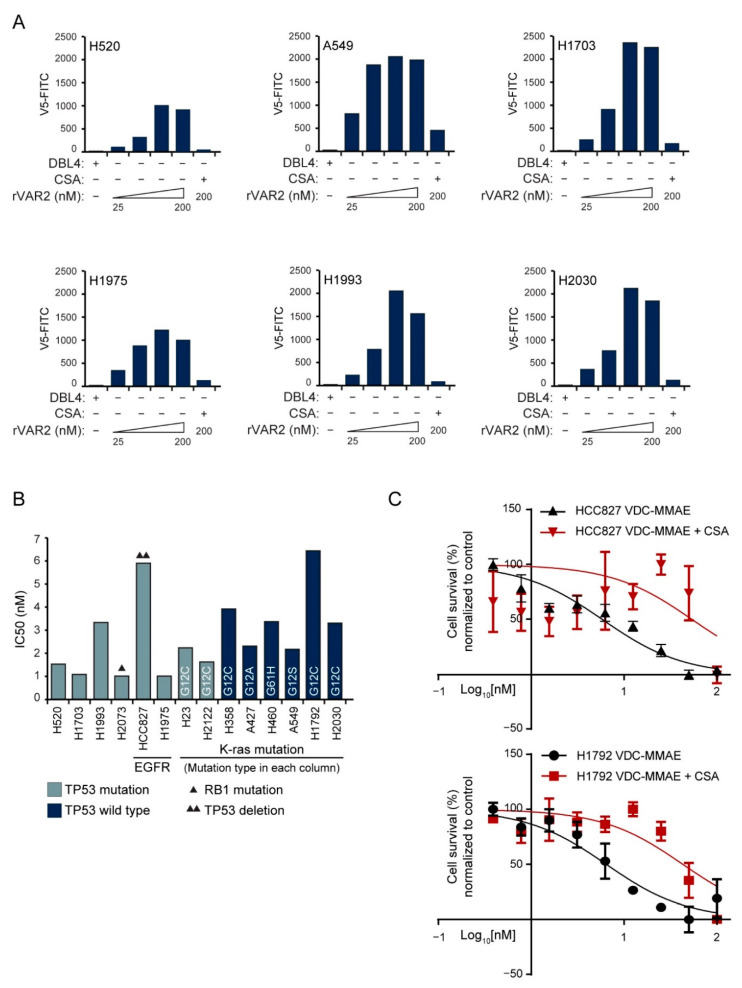
**The** rVAR2 binding and VDC killing assay: (**A**) relative mean fluorescence intensity (MFI) of lung cancer cells, incubated with recombinant control protein (DBL4) or VAR2CSA (rVAR2) as indicated, and detected by flow cytometry using anti-V5-FITC; (**B**) the column graph displays IC50 kill values of VDC-MMAE compound on 14 lung cancer cell lines. Types of KRAS mutation are provided in the column of each cell line; (**C**) VDC-MMAE killing assay with and without CSA competition in two NSCLC cell lines; HCC827 (upper panel) and H1792 (lower panel). Abbreviations: rVAR2: recombinant malaria protein VAR2CSA; VAR2CSA: variant surface antigen 2-CSA; VDC: rVAR2 drug conjugate; CSA: chondroitin sulfate A; MMAE: monomethyl auristatin E; FITC: fluorescein isothiocyanate; IC50: inhibitory concentration 50.

We next analyzed a panel of six NSCLC cell lines for oncofetal CS expression by flow cytometry. Using recombinant V5-tagged control protein (DBL4) as the control, we observed a concentration-dependent binding of the rVAR2 protein to oncofetal CS in all NSCLC cell lines tested, saturating at 100 nM rVAR2 (Figure 2A). The binding of rVAR2 to the NSCLC cells could be competed by purified CSA, demonstrating specificity of the rVAR2-oncofetal CS interaction (Figure 2A). Next, we tested the sensitivity of 14 NSCLC cell lines with and without KRAS mutations to the oncofetal CS targeting rVAR2-drug conjugate VDC-MMAE. All NSCLC cell lines were sensitive to VDC-MMAE treatment with IC50 values ranging from 1 to 6.5 nM (Figure 2B). Importantly, VDC-MMAE cytotoxicity was reduced by CSA competition with 7–9 times higher IC50 values, indicating drug-specificity towards oncofetal CS (Figure 2C). These data show that both KRAS wild-type and mutant NSCLC cells are positive for oncofetal CS and sensitive to VDC-MMAE in vitro.

### 3.4. VDC-MMAE Inhibits Growth of NSCLC Tumors In Vivo

**Figure 3:**
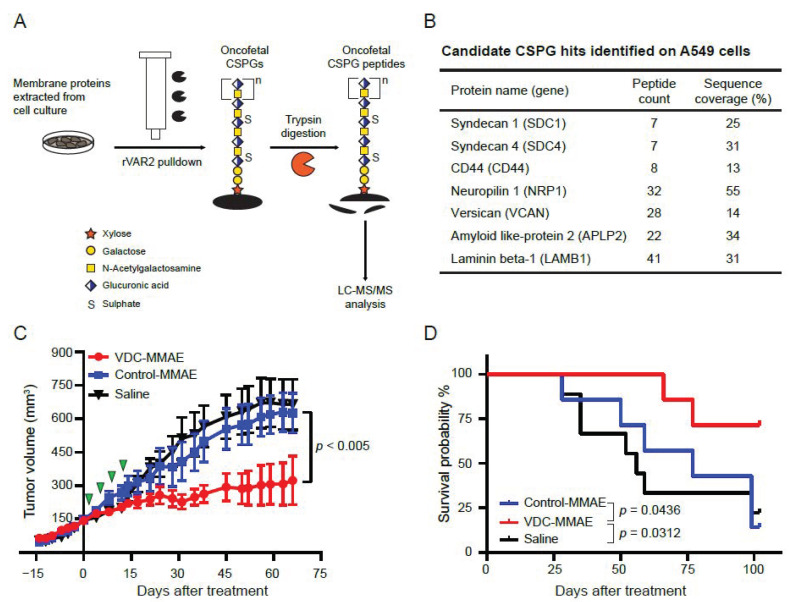
Proteomics analysis on A549 cells and effect of VDC-MMAE in A549 xenografts: (**A**) a workflow diagram for affinity purification and mass spectrometry analysis. (**B**) Oncofetal CS-modified CSPGs identified with rVAR2 pulldown and mass spectrometry on A549 cell line. (**C**) Comparison of tumor growth between VDC-MMAE and control groups. Treatment was administered intravenously twice per week for two weeks (green arrow heads) by intravenous injection in the tail vein as indicated. (**D**) Survival curve of VDC-MMAE and control groups from (**C**). Abbreviations: CSPG: chondroitin sulphate proteoglycan; rVAR2: recombinant malaria VAR2CSA protein; VAR2CSA: variant surface antigen 2-CSA; VDC: rVAR2 drug conjugate; MMAE: monomethyl auristatin E.

We next investigated the sensitivity of established in vivo NSCLC xenograft tumors to VDC-MMAE. A549 is one of the best characterized NSCLC cell lines with a homozygous KRAS G12S mutation and extensively used for xenograft studies in mice [26,27]. We first confirmed the expression of known CSPGs in A549 cells by an affinity purification and mass spectrometry workflow (Figure 3A). The analysis identified multiple proteoglycans with oncofetal CS modifications including SDC1, SDC4, CD44, and Versican (Figure 3B). We next established A549 xenograft tumors in mice followed by treatment with 15 mg/kg of VDC-MMAE twice a week for two weeks. Contrary to vehicle and control-MMAE treatments, VDC-MMAE effectively inhibited tumor growth (Figure 3C) and prolonged survival of the mice (Figure 3D). These results show that oncofetal CS-positive NSCLC tumors can be targeted by VDC-MMAE in vivo independent of KRAS-mutation status.

## 4. Discussion

ADCs are approved for the treatment of several cancer types and represent a promising approach to improve the prognosis of advanced NSCLC. ADC targets that are present both on tumor cells and stromal compartments represent a more effective therapeutic opportunity.

Informative biomarkers are important for disease management and are most commonly assessed by IHC and/or genomics-based approaches. IHC biomarker stainings are affordable and widely available, such as PD-L1 stainings that offer fast laboratory turnaround time, which is essential for patients with highly progressing cancers. Gene sequencing facilities able to perform genomic interrogations are often located in centers and/or require shipment of biospecimens, which can be time-demanding and costly. These approaches are challenged by fast tumor progression in NSCLC that associate with pleural effusion, brain or liver metastases, or cachexia that limits the therapeutic window. Therefore, patients often receive no active therapy and supportive care that are under-represented in clinical trials. A large portion of NSCLCs are not linked to any oncogenic drivers. When an oncogene-based therapy is identified, a number of these subtypes have a prevalence of less than 5%. Additionally, a recent analysis showed that less than 50% of patients with metastatic NSCLC do not have their tumors tested broadly for genomic aberrations, missing treatment opportunities with small molecules [28].

We previously described that an evolutionarily refined parasite–host anchor protein, VAR2CSA, derived from *P. falciparum* malaria parasites, specifically binds to an oncofetal CS modification on a subset of cancer-associated proteoglycans with high affinity and specificity [16,17,18,19]. In the present study, we added that oncofetal CS was highly expressed in stage I and II tumors and associated with poor OS and DFS in NSCLC patients. We observed strong oncofetal CS expression in both pericellular and extracellular matrix compartments with no correlation to EGFR or KRAS mutation. Accordingly, there is a potential opportunity for broad therapeutic application towards NSCLC with no limitation to certain subsets. Notably, we frequently observed high oncofetal CS expression in NSCLC with squamous cell histology, suggesting that oncofetal CS-derived therapeutics can be a useful therapeutic option for lung squamous cell carcinomas where no targeted therapy is otherwise available. In agreement with this, we observed VDC-MMAE sensitivity in all advanced NSCLC cell lines tested regardless of gene mutation status, histology, or oncogene expression. Hence, oncofetal CS constitutes a biomarker applicable for both targeted therapy as well as prognostic stratification of NSCLC.

High oncofetal CS expression is associated with poor DFS in NSCLC patients [16]. This is in line with other solid tumor malignancies such as melanoma and muscle-invasive bladder cancer [16,19]. Interestingly, elevated CHST11 expression, the enzyme required for CSA 4-*O*-sulfation, is also associated with poor DFS in three independent lung cancer cohorts [16]. A higher proportion of highly-sulfated CS was observed in the highly-metastatic LM660H11 lung cancer cell line, as compared to the P29 cell line with low metastatic potential [29]. Notably, preincubating Lewis lung cancer cells with rVAR2 lectins before intra-cardiac injection, strongly impeded the metastatic seeding in distant organs as compared to control groups [18]. Additionally, rVAR2-coated beads can capture circulating lung cancer cells with high specificity and efficiency from patient blood samples, as well as A549 cells spiked into healthy donor blood, even after transforming them into mesenchymal phenotype to resemble a step within metastatic cascade [30]. These studies indicate that in addition to early-stage NSCLC, oncofetal CS modifications are also expressed in later stages of lung cancer.

Curative intent lung resection surgery is the only effective therapy that results in long-term survival, but 55% of NSCLC patients experience recurrence [31]. For advanced disease, the current treatment paradigm includes immunotherapy, chemotherapy, and oncogenic driver-based targeted therapy. Recently, ADCs are being developed for NSCLC. Although tremendous resources are put into the search for new, targeted therapeutic options, no clear improvement in OS has been seen in combination with anti-angiogenic therapy in NSCLC [32] and there are no consistent results from tyrosine kinase inhibitor adjuvant studies [33]. Cancers with a high mutation burden, such as smoking-related lung cancer, require high sequencing capacity to reach sufficient sensitivity for the identification of low-frequency driver genes [34]. Targeting broadly expressed cancer-specific post-translational modifications such as oncofetal CS is an attractive strategy as these modifications are often expressed redundantly on different proteoglycans and do not depend on single gene alterations [35,36], making treatment resistance less likely to occur.

Aberrant glycosylation and expression of CSPGs are common in tumor initiation, progression, and prognosis [8,16,37]. Our oncofetal CS pulldown experiment on the A549 cell line identified a limited repertoire of oncofetal CS-modified CSPGs (Figure 3B). Among them, SDC1 expression is reportedly associated with NSCLC patient survival [38], independent of EGFR expression [39]. Additionally, high serum levels of SDC1, measured by ELISA, is an independent, poor-prognostic classifier in lung cancer patients [40]. Additionally, pre-treatment serum SDC1 levels can predict outcome in small cell lung cancer patients treated with platinum-based chemotherapy [41]. Recently, we validated the novel CS-glycosylation site on human SDC4 protein, which can be modified by CS chains at the attachment sites Ser39, Ser61, and Ser63 [25]. CD44 (also known as CSPG8) is another major CSPG identified in our analysis that is involved in tumorigenesis. CD44 inhibition attenuates EGFR signaling and enhances cisplatin sensitivity in EGFR wild-type NSCLC [42]. Additionally, CD44 promotes PD-L1 expression and its tumor-intrinsic function in both breast and lung malignancies [43]. High stromal expression of Versican correlates with poor tumor differentiation, disease recurrence, advanced tumor stage, and lymph node metastases [44]. Neuropilin 1 (NRP1) modulates TGF-β1-induced epithelial-mesenchymal transition in NSCLC [45], and dual-targeting of EGFR and NRP1 attenuates resistance to EGFR-targeted antibody therapy in KRAS-mutant NSCLC [46]. NRP1 expression correlates with radio-resistance [47], and NRP1 antagonism in human cancer cells inhibits migration and enhances chemosensitivity [48,49]. In our study, all NSCLC cells were effectively killed by VDC-MMAE in low-nM concentration. The A549 in vivo data confirmed that VDC-MMAE can effectively inhibit growth of oncofetal CS-positive NSCLC tumors and extend survival. We did not observe immune-related side effects or organ toxicity in this study or in our previous studies [16,18,19]. Based on dihydrodiol dehydrogenase (DDH) enzyme expression, Chen et al. showed that A549 is one of the most cisplatin-insensitive cell lines [50]. Indeed, VDCs have also shown promising results in cisplatin-resistant bladder tumors [19]. These observations support the idea that patients progressing after platinum-based therapy might still be eligible for oncofetal CS targeting regimens.

Limitations to our study include that our cohort represents a large, early-stage cancer cohort that provides a reasonable basis with both stroma and tumor compartment detailed assessments for the expression landscape of glycosaminoglycan. However, advanced NSCLC cases might represent a slightly different pattern. Thus, additional independent clinical prevalence studies in advanced NSCLC are needed to validate our findings in these patient groups.

## 5. Conclusions

In conclusion, our findings identify oncofetal CS as an independent prognosticator in early-stage NSCLC and a potential actionable therapeutic target in advanced NSCLC. Accordingly, our findings support a rationale for exploring oncofetal CS targeting opportunities in NSCLC.

## Figures and Tables

**Table 1:** Relationship between oncofetal CS expression and clinicopathological parameters in 539 NSCLC cases by immunohistochemistry.

Characteristics	Oncofetal CS Expression		*p*-Value
	High	Low	
Age			
≤70 years (*n* = 351)	91 (26%)	260	0.664
>70 years (*n* = 188)	52 (28%)	136	-
Gender			
Female (*n* = 274)	64 (23%)	210	0.090
Male (*n* = 265)	79 (30%)	186	-
Stage			
Stage I (*n* = 318)	81 (25%)	237	0.450
Stage II (*n* = 187)	55 (29%)	132	-
Missing (*n* = 34)	7 (21%)	27	-
Histology			
ADC (*n* = 374)	70 (19%)	304	<0.001
SCC (*n* = 152)	70 (46%)	82	-
Others (*n* = 13)	3 (23%)	10	-
Smoking			
Smoker (*n* = 472)	132 (28%)	340	0.044 *
Non-smoker (*n* = 55)	7 (13%)	48	-
Missing (*n* = 12)	9 (75%)	3	-

Abbreviations: NSCLC: non-small cell lung cancer; CS: chondroitin sulfate; ADC: adenocarcinoma; SCC: squamous cell carcinoma; *p*-values were calculated by the Chi-square test; * Chi-square results may be invalid due to small no. in this sub-table.

**Table 2:** Univariable Cox regression analyses of overall and disease-free survival.

Characteristics		Median OS (Months)			Median DFS (Months)		
		Oncofetal CS		*p*-Value	Oncofetal CS		*p*-Value
		High	Low		High	Low	
Age	Age ≤70	76	80.3	0.26	25.8	78.1	0.04
	Age >70	44.9	57.9	0.23	39	55.4	0.08
Gender	Male	44.3	69.8	0.03	25.4	67.6	0.01
	Female	76	80.3	0.96	39	61.8	0.29
Stage	Stage I	58	77.5	0.16	47	72	0.09
	Stage II	45	52.2	0.39	20	53	0.03
Histology	ADC	45	68	0.18	23	55.4	0.01
	SCC	51.6	72	0.37	NR	NR	0.01
	Others	NR	51	0.61	11.7	55.4	0.98

Abbreviations: ADC: adenocarcinoma; SCC: squamous cell carcinoma; OS: overall survival; DFS: disease-free survival; NR: not reached, which means 50% of the patients were alive or 50% of the patients’ tumor did not recur by this time.

**Table 3:** Multivariable Cox regression analyses of overall and disease-free survival.

Characteristics	OS			DFS		
	HR	95% CI	*p*-Value	HR	95% CI	*p*-Value
Age	1.50	1.15–1.96	<0.01	1.09	0.81–1.46	0.58
Gender	0.85	0.64–1.11	0.23	0.94	0.70–1.26	0.68
Histology	0.96	0.74–1.24	0.77	0.60	0.44–0.83	<0.01
Stage I vs. II	0.74	0.57–0.96	0.03	0.63	0.47–0.84	<0.01
Smoking	0.87	0.53–1.43	0.59	0.68	0.40–1.15	0.15
Oncofetal CS Expression	1.26	0.93–1.69	0.13	1.76	1.32–2.48	<0.01

Abbreviations: CI: confidence interval; HR: hazard ratio; OS: overall survival; DFS: disease-free survival; CS: chondroitin sulfate.

## Data Availability

No new data were created or analyzed in this study. Data sharing is not applicable to this article.

## Supplementary Materials

The following are available online at https://www.mdpi.com/article/10.3390/cancers13174489/s1. Figure S1: Study design and patient outcome of surgically resected NSCLC cohort. Table S1: Relationship between oncofetal CS expression and EGFR/KRAS mutation status.
