# Supplementary material for: Oncofetal Chondroitin Sulfate Is a Highly Expressed Therapeutic Target in Non-Small Cell Lung Cancer
Source: Cancers (Basel). 2021 Sep 6;13(17):4489. doi: 10.3390/cancers13174489 (PMC8430715; doi:10.3390/cancers13174489)
Supplement: Supplementary file 1 [file cancers-13-04489-s001.zip › cancers-1317292-supplementary.pdf]

*Supplementary Materials*

# Oncofetal Chondroitin Sulfate Is a Highly Expressed Therapeutic Target in Non-Small Cell Lung Cancer

**Htoo Zarni Oo, Zoltan Lohinai, Nastaran Khazamipour, Joey Lo, Gunjan Kumar, Jessica Pihl, Hans Adomat, Noushin Nabavi, Hakhamanesh Behmanesh, Beibei Zhai, Robert Dagil, Swati Choudhary, Tobias Gustavsson, Thomas M. Clausen, Jeffrey D. Esko, Jeffrey W. Allen, Michael A. Thompson, Nhan L. Tran, Judit Moldvay, Balazs Dome, Ali Salanti, Nader Al-Nakouzi, Glen J. Weiss and Mads Daugaard**

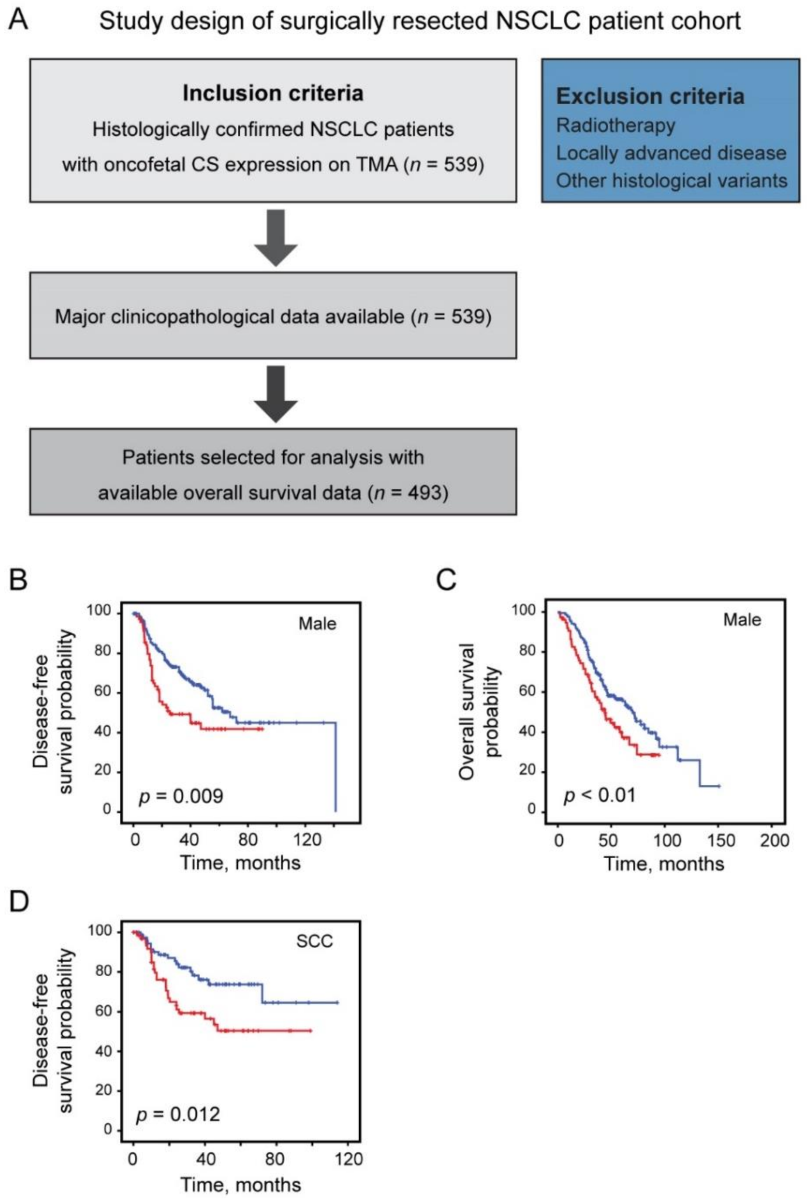


**Figure S1.** Study design and patient outcome of surgically resected NSCLC cohort. (**A**) Study design of surgically resected NSCLC patient cohort; (**B**) survival estimates of DFS in men; (**C**) OS in men and (**D**) DFS in squamous cell carcinoma (SCC) histology. Red color bar: high oncofetal CS expression; blue: low expression. Abbreviations: NSCLC: non-small cell lung cancer; CS: chondroitin sulfate; TMA: tissue microarray.

**Table S1.** Relationship between oncofetal CS expression and EGFR/KRAS mutation status.

| **Mutation Status** | **Oncofetal CS Expression** | | ***p*-Value** |
|---|---|---|---|
| | **High** | **Low** | |
| KRAS mut and EGFR wt | 14 (20%) | 55 | 0.0165 |
| EGFR mut and KRAS wt | 3 (8%) | 36 | |
| EGFR and KRAS wt | 57 (28%) | 145 | |

Abbreviations: mut: mutant; wt: wild type; KRAS: KRAS Proto-Oncogene; EGFR: Epidermal Growth Factor Receptor; *p*-value was calculated by the Chi-square test.

© 2021 by the authors. Licensee MDPI, Basel, Switzerland. This article is an open access article distributed under the terms and conditions of the Creative Commons Attribution (CC BY) license (http://creativecommons.org/licenses/by/4.0/).
